# Supplementary material for: Multi‐dataset identification of innovative feature genes and molecular mechanisms in keratoconus
Source: J Cell Mol Med. 2024 Sep 19;28(18):e70079. doi: 10.1111/jcmm.70079 (PMC11412914; doi:10.1111/jcmm.70079)
Supplement: Supplementary file 2 — Material S2. [file JCMM-28-e70079-s002.zip › 7.GSEA analysis/5.RNF217-AS1/KEGG_ONE_CARBON_POOL_BY_FOLATE.html]

Details for gene set KEGG\_ONE\_CARBON\_POOL\_BY\_FOLATE[GSEA]

|  || Dataset | RNF217-AS1.group.cls#h\_versus\_l |
| Phenotype | group.cls#h\_versus\_l |
| Upregulated in class | l |
| GeneSet | KEGG\_ONE\_CARBON\_POOL\_BY\_FOLATE |
| Enrichment Score (ES) | -0.5276677 |
| Normalized Enrichment Score (NES) | -1.4682741 |
| Nominal p-value | 0.06809338 |
| FDR q-value | 0.29111627 |
| FWER p-Value | 0.805 |
Table: GSEA Results Summary

  

Fig 1: Enrichment plot: KEGG\_ONE\_CARBON\_POOL\_BY\_FOLATE      
 Profile of the Running ES Score & Positions of GeneSet Members on the Rank Ordered List

  

| SYMBOL | TITLE | RANK IN GENE LIST | RANK METRIC SCORE | RUNNING ES | CORE ENRICHMENT || 1 | AMT | na | 7860 | 0.131 | -0.1613 | No |
| 2 | SHMT2 | na | 16383 | 0.005 | -0.4044 | No |
| 3 | MTHFD2 | na | 17185 | -0.003 | -0.4258 | No |
| 4 | GART | na | 19639 | -0.027 | -0.4833 | No |
| 5 | SHMT1 | na | 20561 | -0.036 | -0.4922 | No |
| 6 | MTHFR | na | 21453 | -0.045 | -0.4956 | Yes |
| 7 | MTHFS | na | 22031 | -0.051 | -0.4869 | Yes |
| 8 | ATIC | na | 23447 | -0.067 | -0.4942 | Yes |
| 9 | MTHFD1 | na | 23712 | -0.071 | -0.4665 | Yes |
| 10 | MTFMT | na | 25200 | -0.094 | -0.4630 | Yes |
| 11 | DHFR | na | 26197 | -0.110 | -0.4369 | Yes |
| 12 | MTR | na | 27079 | -0.127 | -0.3990 | Yes |
| 13 | MTHFD2L | na | 27230 | -0.131 | -0.3385 | Yes |
| 14 | MTHFD1L | na | 27681 | -0.141 | -0.2817 | Yes |
| 15 | FTCD | na | 30347 | -0.218 | -0.2503 | Yes |
| 16 | TYMS | na | 31610 | -0.269 | -0.1530 | Yes |
| 17 | ALDH1L1 | na | 34038 | -0.489 | 0.0199 | Yes |
Table: GSEA details [plain text format]

  

Fig 2: KEGG\_ONE\_CARBON\_POOL\_BY\_FOLATE      
 Blue-Pink O' Gram in the Space of the Analyzed GeneSet

  

Fig 3: KEGG\_ONE\_CARBON\_POOL\_BY\_FOLATE: Random ES distribution      
 Gene set null distribution of ES for **KEGG\_ONE\_CARBON\_POOL\_BY\_FOLATE**
